# Supplementary figures and images for: Silver Nanoantibiotics Display Strong Antifungal Activity Against the Emergent Multidrug-Resistant Yeast Candida auris Under Both Planktonic and Biofilm Growing Conditions
Source: Front Microbiol. 2020 Jul 28;11:1673. doi: 10.3389/fmicb.2020.01673 (PMC7399222; doi:10.3389/fmicb.2020.01673)

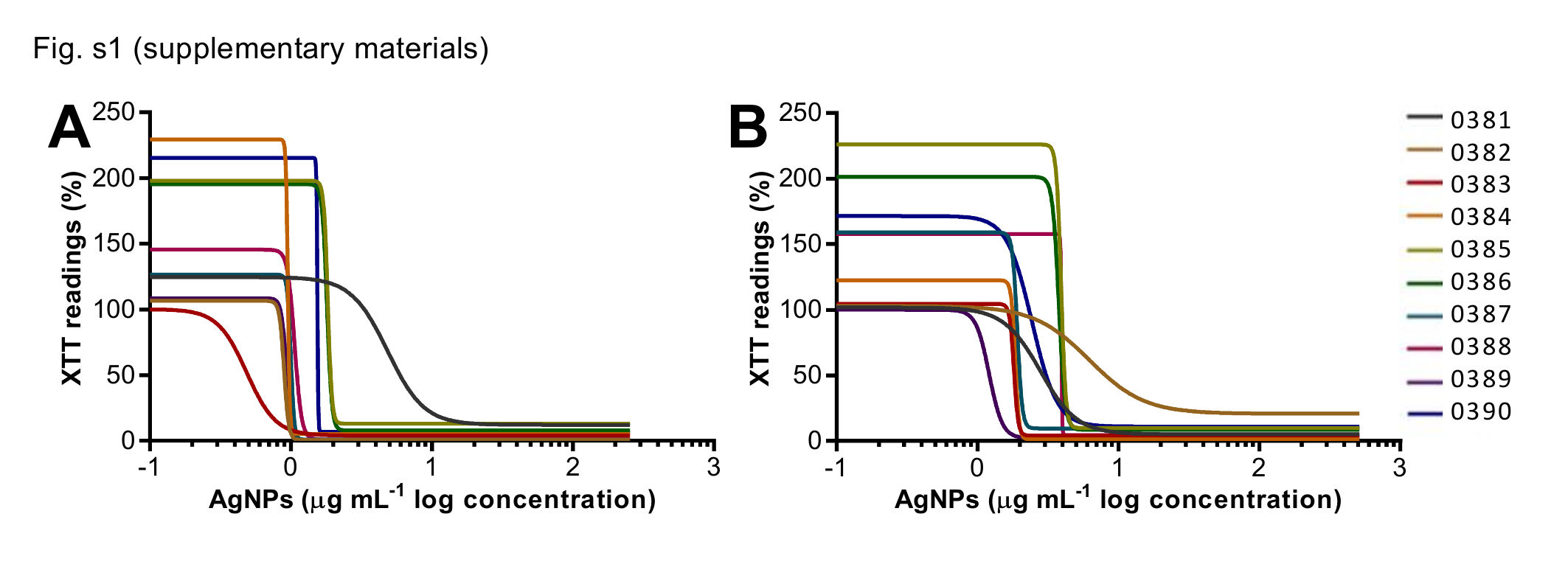

Supplement: SUPPLEMENTARY FIGURE S1 — Antibiofilm activity of AgNPs against the different C. auris strains. The dose-response curve shows that AgNPs display potent inhibitory activity against all the different C. auris strains included in this study, including inhibition of biofilm formation (panel A) and against preformed biofilms (panel B). [file Image_1.TIF]

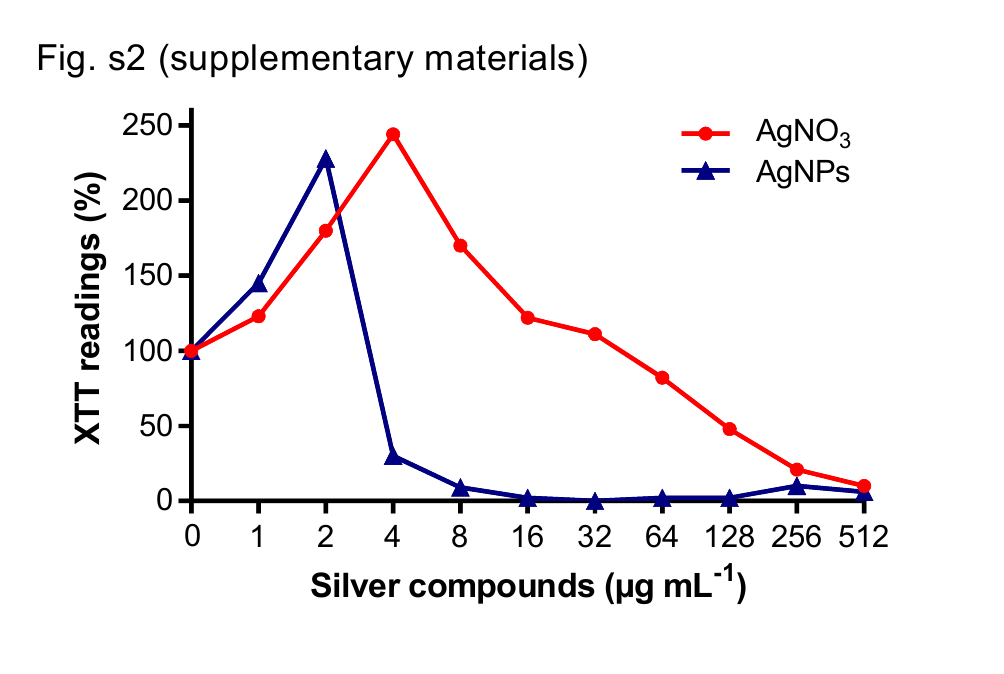

Supplement: SUPPLEMENTARY FIGURE S2 — Silver compounds exhibit a paradoxical effect on C. auris biofilms at low concentrations. Incubation in the presence of low subinhibitory concentrations of AgNO3 and AgNPs lead to an increase in biofilm activity (XTT readings) for biofilms formed by C. auris strain #0390 strain. In contrast to silver ions, this effect rapidly disappears in the case of AgNPs, turning into potent inhibitory activity at still relatively low concentrations. [file Image_2.TIF]

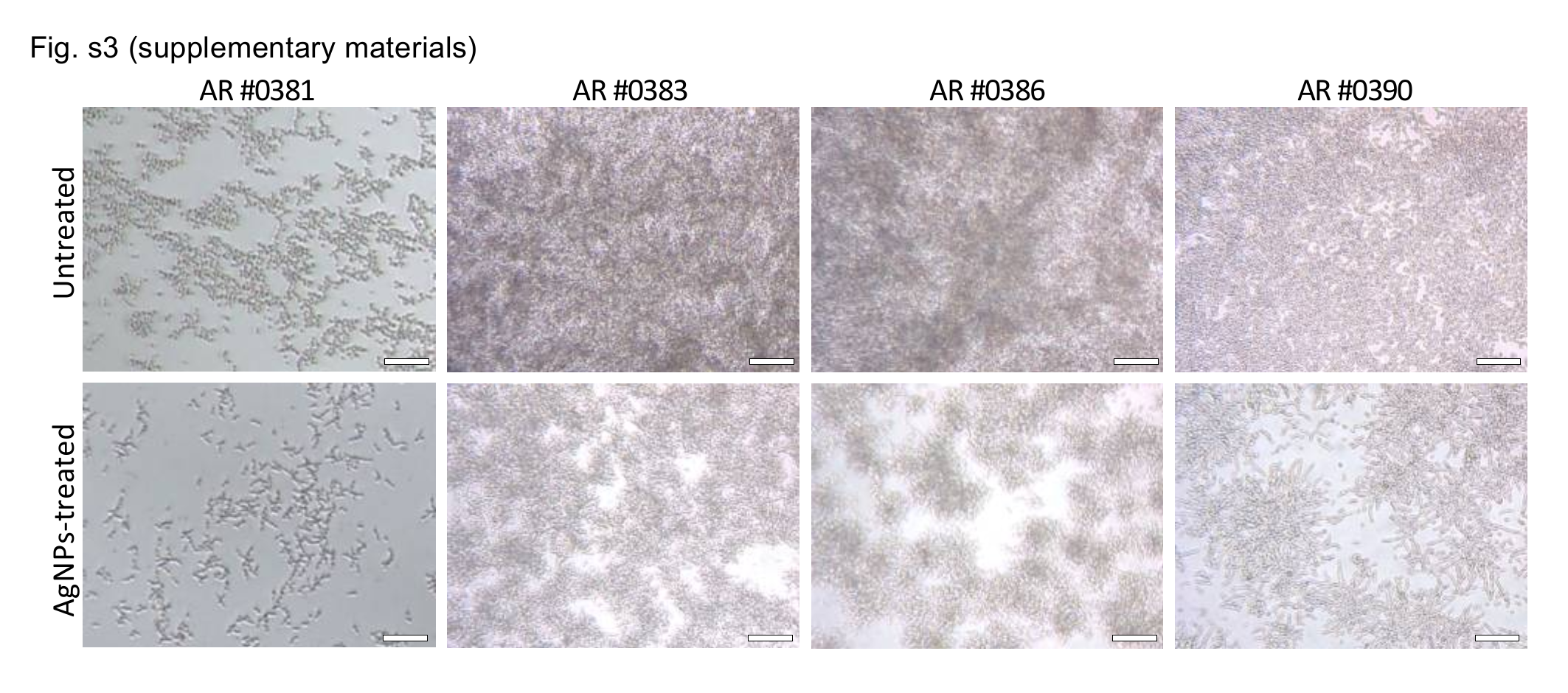

Supplement: SUPPLEMENTARY FIGURE S3 — AgNPs reduce the biofilm formation in C. auris. Optical microscopy images reveal that subinhibitory concentrations AgNPs reduce the ability of C. auris to form biofilms – as seen in the reduced area of biofilm surface coverage – when compared with their respective untreated controls. [file Image_3.TIF]

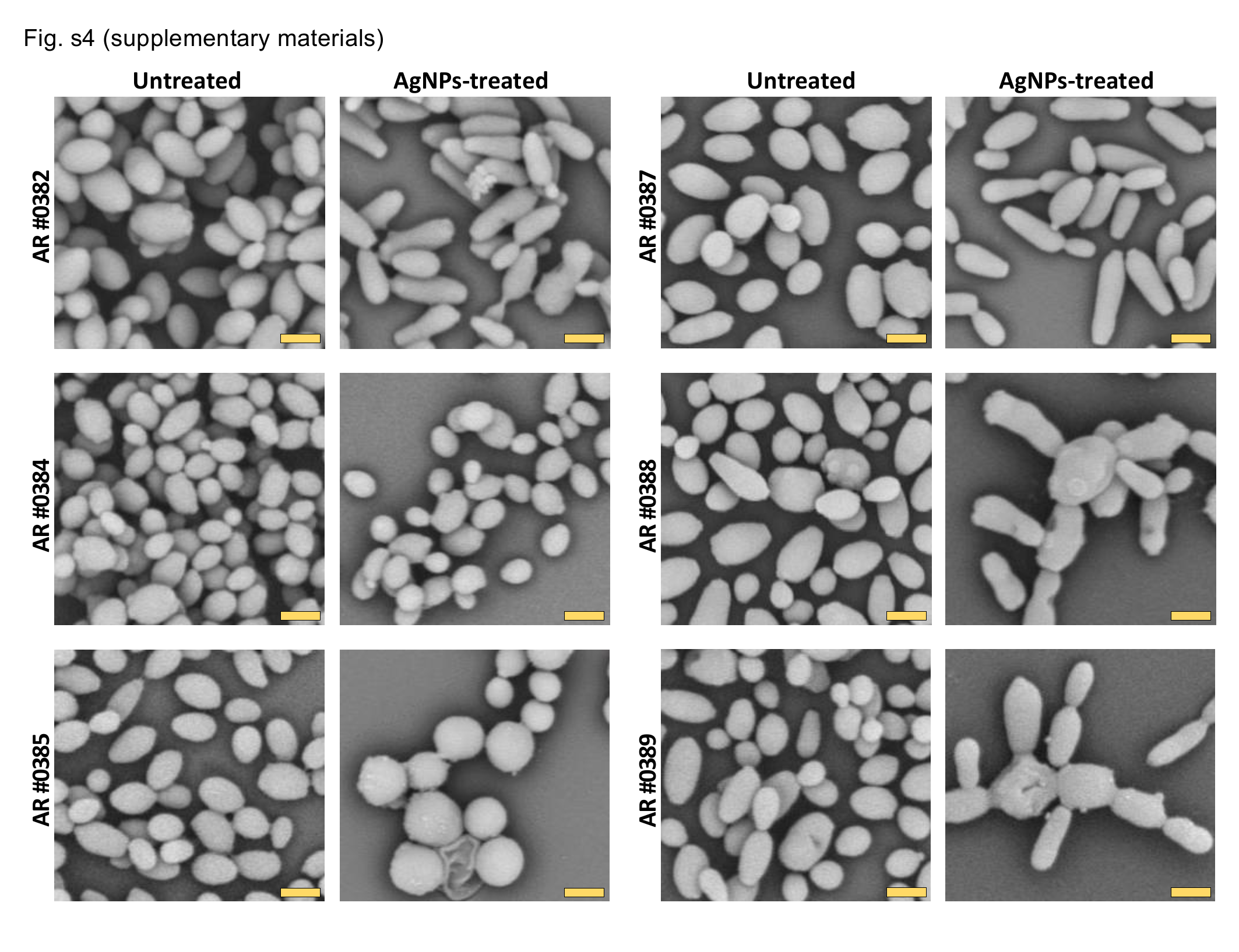

Supplement: SUPPLEMENTARY FIGURE S4 — Ultrastructure analysis of the C. auris biofilms. SEM images reveal that subinhibitory concentrations of AgNPs reduce the biofilm formation on all C. auris strains. Also, AgNPs negatively alter the shape and size in some C. auris strains (#0382, #0385, #0387, #0388, and #0389 strains). The effect on morphology is clade-related. Scale bar = 2 μm. [file Image_4.TIF]
